# The added value of eye-tracking in diagnosing dyscalculia: a case study

**DOI:** 10.3389/fpsyg.2013.00679

**Published:** 2013-10-01

**Authors:** Sietske van Viersen, Esther M. Slot, Evelyn H. Kroesbergen, Jaccoline E. van't Noordende, Paul P. M. Leseman

**Affiliations:** Department of Cognitive and Motor Disabilities, Utrecht UniversityUtrecht, Netherlands

**Keywords:** dyscalculia, number sense, eye-tracking, number line, mapping, diagnostic procedures

## Abstract

The present study compared eye movements and performance of a 9-year-old girl with Developmental Dyscalculia (DD) on a series of number line tasks to those of a group of typically developing (TD) children (*n* = 10), in order to answer the question whether eye-tracking data from number line estimation tasks can be a useful tool to discriminate between TD children and children with a number processing deficit. Quantitative results indicated that the child with dyscalculia performed worse on all symbolic number line tasks compared to the control group, indicated by a low linear fit (*R*^2^) and a low accuracy measured by mean percent absolute error. In contrast to the control group, her magnitude representations seemed to be better represented by a logarithmic than a linear fit. Furthermore, qualitative analyses on the data of the child with dyscalculia revealed more unidentifiable fixation patterns in the processing of multi-digit numbers and more dysfunctional estimation strategy use in one third of the estimation trials as opposed to ~10% in the control group. In line with her dyscalculia diagnosis, these results confirm the difficulties with spatially representing and manipulating numerosities on a number line, resulting in inflexible and inadequate estimation or processing strategies. It can be concluded from this case study that eye-tracking data can be used to discern different number processing and estimation strategies in TD children and children with a number processing deficit. Hence, eye-tracking data in combination with number line estimation tasks might be a valuable and promising addition to current diagnostic measures.

The present study investigated the added value of number line estimation tasks combined with eye-tracking data for the diagnostic procedure of dyscalculia. Although little is known about the validity or utility of eye movements as a diagnostic measure of mathematical difficulties, eye-tracking seems a valid instrument to examine the underlying processes in number line estimation (Schneider et al., 2008; Heine et al., 2010; Sullivan et al., 2011). The use of eye movements in combination with number line tasks as a diagnostic instrument might provide important insights in the processing of (non-)numerical information and possible underlying deficits in children with dyscalculia. Current diagnostic measures are mainly focused on the mathematical achievement level and therefore less adequate for identifying a cognitive deficit in numerical processing skills. The eye-tracking data can reveal underlying processes and could also lead toward more knowledge about the use or absence of number processing and estimation strategies and their detectability in children with dyscalculia.

Developmental Dyscalculia (DD) is defined as a learning disability characterized by severe mathematical problems. Children with DD often show difficulties in representing and manipulating numerical information non-verbally and spatially, in the automation of arithmetic facts, and in executing arithmetic procedures (prevalence 3–6%; Rotzer et al., 2009; De Visscher and Noel, 2012). Concretely, children with DD often display difficulties with for example approximation, counting sequences, and subitizing (e.g., Butterworth et al., 2011; Mazzocco et al., 2011; Desoete et al., 2012; Geary et al., 2012). DD has been linked to a core deficit in number sense, i.e., the spatial processing of (number) magnitude information (Dehaene, 1997; Wilson and Dehaene, 2007), possibly caused by impaired functioning of the intra parietal sulcus (IPS; Kucian et al., 2006; Rotzer et al., 2008). According to Dehaene (2001), number sense refers to the “fundamental ability to mentally represent and manipulate numerosities on a mental ‘number line”’ (p. 17), a line along which numerical magnitudes are represented in ascending order with respect to their magnitude (Moeller et al., 2009b). The development of the mental number line is based on the ability to map number symbols onto their corresponding non-symbolic magnitude (Kolkman et al., 2013). Between the ages of 3.5 and 8 years, typical children become increasingly aware of the concept of numerosity, i.e., the understanding that number symbols are connected to quantities (Kolkman et al., 2013). This change results from transcoding processes, enabling the child to translate information from non-symbolic to symbolic format and vice versa, providing the number symbols with a non-symbolic magnitude meaning (Dehaene, 2001). In line with Kolkman et al. (2013) we refer to these skills as “mapping skills.”

Number line tasks are often used to measure mapping skills (e.g., Schneider et al., 2008; Kolkman et al., 2013). In these tasks, children have to estimate the position of a given number on a horizontal number line from 0 to 10, 0 to 100, or 0 to 1000. Previous research has shown that many children with DD have difficulties with estimating the correct positions of numbers on a number line (e.g., Kucian et al., 2006), probably caused by a deficient development of the mapping skills. Young children that have not fully developed mapping skills also tend to make inaccurate logarithmic placements instead of linear placements on a number line (Booth and Siegler, 2006). For example, Moeller et al. (2009b) found that young children tend to overestimate single-digit numbers, using up to half of the number line and placing all two-digit numbers in the remaining space. Larger and less familiar numbers also lead to a decrease in accuracy (Ebersbach et al., 2008). This indicates that the development of the mental number line in young children (kindergarten age) is typically characterized by a logarithmic representation, meaning that the distance between the magnitudes of numbers at the low end of the range is exaggerated and the distance between magnitudes of numbers in the middle and upper ends of the range are minimized (Siegler and Booth, 2004; Kucian et al., 2006). When number representations become more precise, a shift from a logarithmic to a linear ruler representation has been observed in typically developing (TD) children. The timing of this shift depends on the number range of the number line; the linear representation is observed earlier in small number ranges than in larger number ranges (Siegler and Booth, 2004; Booth and Siegler, 2006; Opfer and Siegler, 2007; Siegler et al., 2009; Friso-van den Bos et al., under review). Empirical evidence suggests that 10-year-old children with dyscalculia display number representations at the level of 5-year-old TD children (Piazza et al., 2010), although there is also evidence showing higher linear than logarithmic fit in children with math problems (e.g., Van't Noordende and Kolkman, 2013).

Concerning the performance of children with DD on number line tasks, it is important to note that a deficit in number sense is not the only factor that might explain poor results on the estimation of numbers. Number line tasks require specific working memory abilities such as visuospatial processing and visuospatial storage (Kolkman et al., under review). Children with DD often show working memory deficits, especially in the visuospatial domain (Kroesbergen et al., 2007; Toll et al., 2011; Geary et al., 2012; Passolunghi and Mammarella, 2012; De Weerdt et al., 2013). Poor spatial working memory processes might partly underlie the mapping deficit, inhibiting the formation of a mental number line as well as the storage and retrieval of arithmetic facts (Rotzer et al., 2009; Kolkman et al., under review). Consequently, working memory ability was included in this study as a background variable, as it may form an additional explanation for the low performance and strategy use of the child with DD on the number line task.

## Number processing and estimation strategies

Several theories have been derived from eye-tracking research on the processing and estimation of (non-)symbolic magnitudes and single- and multiple-digit numbers. These theories describe observable strategies, indicated by eye movement patterns, in children with typically developing math skills. The present study will examine whether a child with dyscalculia displays atypical number processing and estimation strategies, which would confirm a deficit in (non-)numerical processing skills.

Research on numerical processing pertains solely to the processing of symbolic numbers, represented in Arabic symbols, consisting of two or more digits. Nuerk et al. (2011) provide a definition of multi-digit number processing, stating that processing of multiple-digit numbers depends on the integration or computation of at least two digits to realize a numerical entity. Roughly three strategies can be identified: (a) *holistic* processing, (b) *decomposed parallel* processing, and (c) *decomposed sequential* processing. According to Dehaene et al. (1990), a holistic strategy suggests that, before mental manipulation of numerical information takes place, magnitudes are transformed into distance measures mapped onto a mental number line. Numbers are assumed to be processed as an integrated entity, and not decomposed into tens and units (Dehaene et al., 1990). In contrast, in a broad review on multi-digit processing Nuerk et al. (2011) found evidence that the processing of two-digit numbers always occurs in a decomposed way. Decomposed processing means that numbers are “decomposed” into hundreds, tens, and units. However, there is little evidence about how decomposed processing functions. Poltrock and Schwartz (1984) argued for sequential decomposition, where multi-digit numbers are compared in a sequential digit-by-digit way, starting at the leftmost digit. In contrast, Nuerk and Willmes (2005) argue for parallel decomposition, implying that multi-digit numbers are decomposed and processed in a parallel fashion, considering the value of every digit in relation to the other.

All three strategies can be identified by specific eye movement patterns. As described by Moeller et al. (2009a), holistic processing can be identified by equal fixations at all digits. This way numbers are processed as an integrated entity, resulting in a “pictorial representation” of the number. Decomposed parallel processing is indicated by fixations at the largest entity first (e.g., tens) and less or shorter fixations at the smaller entity (e.g., units), carefully comparing the values of the digits from largest to smallest. Fixations only at the largest entity and (almost) no fixation at the smaller entity, or solely after the child has already started estimating, is indicative of decomposed sequential processing (Moeller et al., 2009a). So far, evidence was found for decomposed parallel processing of two-digit numbers (Moeller et al., 2009a) and three-digit numbers (Korvorst and Damian, 2008) in a general population. To the best of our knowledge, no evidence was found for the holistic and decomposed sequential strategies in eye-tracking data (Moeller et al., 2009a).

Research on estimation of magnitudes and numbers identified several strategies that can be used to estimate numbers on a line. Newman and Berger (1984) and Petitto (1990) stated that children in kindergarten and the first years of primary school were prone to use a *counting-up* or *counting-down* strategy; they start at one end of the line and count up or down in whole units or decades until they reach the target position. Older TD children tend to use a *midpoint* strategy; if the target position is closer to the midpoint of the line than to one of its ends, counting starts at the middle and subsequently up or down from there (Newman and Berger, 1984; Petitto, 1990; Schneider et al., 2008). Ultimately, the use of these strategies is adapted to the target number that has to be estimated (Newman and Berger, 1984). Sullivan et al. (2011) showed that eye-tracking is a valid instrument to measure the use of these strategies. Eye movement patterns have been shown to be also adequate for detecting differences in strategy use (e.g., Schneider et al., 2008; Van't Noordende and Kolkman, 2013). Van't Noordende and Kolkman (2013) studied differences in eye movements between TD children and children with mathematical learning disabilities. They found that children with math learning disabilities use different strategies than children without math learning problems. They make less use of the reference points related to the specific strategies (i.e., begin, midpoint, and end). Moreover, most of their gazes to the reference points are on the midpoint and less on the begin- and endpoint of the line.

The present study compared the eye movements of a child with dyscalculia on a series of number line tasks to those of a control group in an explorative manner, in order to get a first indication of whether eye-tracking data from tasks tapping on number sense can be a useful tool in the diagnostic process of dyscalculia. It was hypothesized that the child with dyscalculia would show more atypical and more unidentifiable eye movement patterns than the control group when processing two-digit and three-digit numbers. In addition, it was hypothesized that the child would show more dysfunctional and more undefined strategies than the control group when estimating numbers on a number line, as she might make less use of reference points (i.e., begin, middle, end) and gaze relatively more at the midpoint (Van't Noordende and Kolkman, 2013). Moreover, it was expected that the eye-tracking data of the child with dyscalculia would indicate that her number representations were still logarithmic rather than linear, resulting in inaccuracy in estimation of multi-digit numbers as indicated by a higher mean percent absolute error than the control group. The data were analyzed using both quantitative and qualitative techniques.

## Methods

### Participants

This case study concerned a 9-year-old Dutch girl (L), who had recently been diagnosed with DD at the diagnostic center of the Faculty of Social and Behavioral Sciences at Utrecht University (i.e., Ambulatorium), according to the three criteria as described by Van Luit et al. (2012). First, the diagnostic tests revealed that L's math abilities were significantly lower than expected based on her cognitive abilities (i.e., discrepancy criterion): L was tested as averagely intelligent, but on the timed math test she obtained a percentile score of 1. Second, L appeared to be substantially behind (i.e., more than 1.5 years) in basic numeracy skills and her level of automation (i.e., downfall criterion). Her numerical skills were comparable with the level of a student in second grade. Third, L did not benefit from the remedial teaching she received since Grade 2 (i.e., didactic resistance criterion).

The control group consisted of 10 TD children, of approximately the same age as L, from one school in the Netherlands (*M*_age_ = 9.1, *SD*_age_ = 0.6). All children performed within the average range on math ability (between the 50 and 75th percentile), as measured by a standardized Dutch complex arithmetic test, and did not have a history of behavioral or learning problems.

### Instruments

Both the control group and L were assessed on a small range of behavioral and cognitive measures in order to compare their number sense, arithmetic and working memory abilities.

#### Mathematics

Recent percentile scores from a complex, standardized school arithmetic test (Janssen et al., 2005) were provided by the teachers of all control children to obtain information on their arithmetic abilities compared to the child with dyscalculia. The test consisted of 50 mathematical word problems and is generally administered at the end of the school year at almost all schools in The Netherlands.

#### Number sense

Three computerized number line tasks were used to measure symbolic and non-symbolic magnitude representations and multiple-digit representations. One non-symbolic number line task (i.e., 0–100) and two symbolic number line tasks (i.e., 0–100, and 0–1000) were included in the test battery, similar to the tasks used by Van't Noordende and Kolkman (2013). The outcomes of the tasks are *R*^2^_lin_ and *R*^2^_log_, indicating the linear and logarithmic fit, which refer to the variance that is explained by a certain function. In addition, the mean percent absolute error for each child is computed, which is obtained by subtracting the correct quantity from the estimate and dividing it by the scale of estimates (e.g., 100 or 1000; Booth and Siegler, 2006).

***Non-symbolic tasks.*** The non-symbolic number line task consisted of magnitudes in the form of dots, representing drops of fuel. The child was told to estimate how far the car could drive with a certain number of “fuel drops” and indicate this distance by placing the lever on the number line. The left side of the number line showed zero dots and the right side of the line showed 100 dots. The task consisted of 33 items.

***Symbolic tasks.*** In the symbolic number line tasks, the child was asked to estimate magnitudes in the form of digits. The left side of the number line showed the number 0, the right side of the line showed to number 100 or 1000, and a random number was depicted beneath the number line in each trial. The child had to verbally repeat the number and then estimate were it would belong on the number line by placing the lever. The 0–100 and 0–1000 tasks both included 33 items.

#### Working memory

L's (working) memory capacity was measured using several subtests of the *Automated Working Memory Assessment* battery (AWMA; Alloway, 2007). Control children were also assessed on two short-term memory subtests (non-word recall and dot matrix). All subtests were discontinued after three incorrect answers. Percentile scores were reported. Verbal short-term memory was measured using digit recall, in which the child recalled increasing series of digits, and non-word recall, in which the child recalled increasing series of non-words. Verbal working memory was measured by the listening recall subtest, in which the child had to answer questions, and after answering a series of questions, recall the first word of each question. Test-retest reliabilities of these subtests are good (Alloway et al., 2009).

Visuospatial short-term memory was measured using the subtest dot matrix, in which the child was shown a 4 × 4 grid of empty white squares, with red dots appearing in one square at a time. The child had to reproduce the sequence of the dot by pointing out the same sequence in an empty grid. The subtest odd-one-out was used to measure visuospatial working memory. This subtest required the child to indicate in increasingly complex sequences which figure out of three was odd, and recall the odd figures in a matrix. Test-retest reliabilities are good (Alloway et al., 2009).

#### Number processing and estimation strategies

The Tobii T60 Eye-Tracker was used to register the eye fixations during the number line tasks. This eye-tracker was installed on a Windows computer, situated in the lab of the university. Recordings of the eye movements, as well as heat maps and gaze plots could be derived from the eye-tracker for the analysis of the fixation patterns.

### Procedure

For L, an intern of the Ambulatorium administered the behavioral and cognitive measures of the assessment battery. A PhD-student administered the number line tasks and the eye-tracking data. Data was gathered within 1 h. For the control group, graduate students visited a school to assess 15 control children, of which 10 were included in the control group. The math test was administered in a classroom setting, the cognitive and number line tasks were all administered individually in a quiet room somewhere in the school. Data was gathered within 1 h for every child.

### Analysis

The data was analyzed both quantitatively and qualitatively. The quantitative analyses focused on the comparison of the performance on the number line tasks and working memory performance between L and the control group. For the number line tasks, the level of performance was indicated by measures of logarithmic and linear fit and the mean percent absolute error. For working memory, the level of performance was indicated by percentiles on two different memory components. The aim of these analyses was to establish the representativeness of the control group and to provide an insight in the specific deficits that L displays. To investigate processing and estimation strategies, qualitative analyses were used to observe and code the eye fixation patterns in the eye-tracking videos. An attempt was made to discern differences in strategy use between L and the control group. Since the use of eye-tracking data in combination with number line tasks is still experimental, no predefined coding schemes were available (i.e., in terms of pixel settings or fixation duration in milliseconds). Consequently, the eye fixation patterns were explored based on descriptive information derived from the literature on possible strategy use in number processing and estimation. Coding was performed by two trained PhD-students with experience in analyzing eye-tracking data. Cohen's kappa was computed to determine the inter rater reliability, which was 0.77 for the processing strategies on both symbolic tasks and 0.64 and 0.71 for the estimation strategies on the 0–100 and 0–1000 symbolic number line tasks.

**Figure 1:**
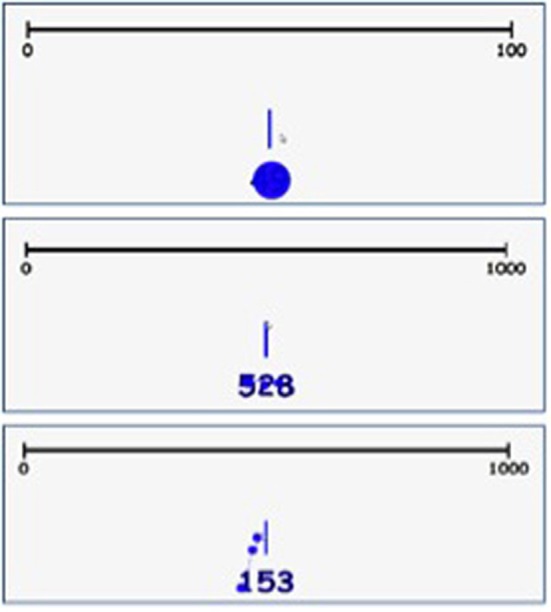
**Examples of the number processing strategies holistic (top), decomposed parallel (middle), and decomposed sequential (bottom)**.

First, the number processing strategies were coded based on the information from the studies of Nuerk et al. (2011) and Moeller et al. (2009a). For each trial, the sequences of eye movements on the given numbers were determined while the child attempted to estimate its place on the number line. As elaborated on before, each strategy can be identified based on a specific fixation pattern. Holistic processing was identified by equal fixation (as determined by an expanding dot indicating fixation location and duration, see Figure 1 for the representative dot sizes per strategy) at all digits at the same time, before the child moved the lever to the number line. Decomposed parallel processing was indicated by fixations starting at the largest entity (e.g., hundreds) and less or shorter fixations progressing toward the smaller entities (e.g., tens), all before the child moved the lever to the number line. Decomposed sequential processing was indicated by a fixation only at the largest entity, which happened before the child moved the lever to the number line, and (almost) no fixation at the smaller entity, which might also occur while the child is already lugging the lever across the number line. If the eye movements showed unclear sequences that did not correspond to any of the predefined strategies, the trial was coded as *undefined*. When the eye movements indicated a pattern that seemed not random but rather atypical, and also did not fit any of the three strategies that were derived from the literature [see “unexpected observed fixation pattern” in Moeller et al. (2009a)], the trial was coded as *other*. Examples of fixation patterns belonging to the different number processing strategies are displayed in Figure 1.

**Figure 2:**
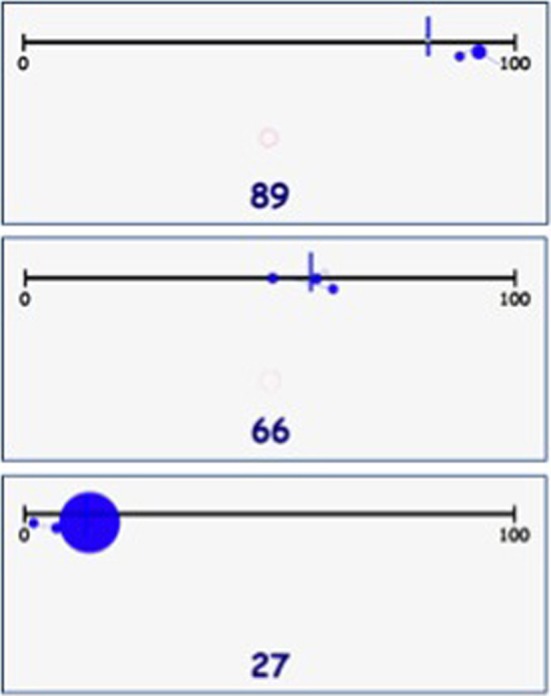
**Examples of the number estimation strategies counting-down/endpoint (top), midpoint (middle), and counting-up/beginpoint (bottom)**.

Second, the number estimation strategies were coded based on studies of Petitto (1990) and Newman and Berger (1984). Every strategy was identified based on the presence (or absence) of a specific fixation pattern. The *counting-up/counting-down* strategy indicated that the begin or the endpoint of the number line was used as reference to estimate a number. The *midpoint* strategy was identified by eye movements that indicated that the (exact) middle of the number line was used as a reference point (i.e., 50 in the 0–100 task and 500 in the 0–1000 task, with a margin of ±5% of the line length). In all three strategies, fixations at the chosen reference point had to be followed by the child step-by-step lugging the lever while estimating the given number. When a child showed fixation patterns that fitted multiple strategies or when it was not possible to discern a specific fixation pattern in the eye movements, the trial was coded as *undefined*. In addition to coding strategy use, it was determined per trial whether the used strategy was functional or dysfunctional. For making this distinction, the number line was divided in four equal quarters, which were each paired with a functional strategy. The *counting-up* strategy was considered functional for numbers between zero and 25(0), the *midpoint* strategy for numbers between 25(0) and 75(0), and the *counting-down* strategy for numbers between 75(0) and 100(0). Since this division seems rather arbitrary, we focused on relative differences between L and the control group in functional vs. dysfunctional strategy use, excluding the unidentifiable trials. Examples of fixation patterns belonging to the different number estimation strategies are displayed in Figure 2.

Differences between L and the control group were tested for significance with the SINGLIMS_ES.exe program, which is specifically designed for comparing a single case score to a control sample mean (Crawford and Howell, 1998; Crawford and Garthwaite, 2002; Crawford et al., 2010). Outcome measures are *p*-values and the *z*_*cc*_-index, which represents an estimate of the average difference, measured in standard deviation units in order to be scale independent, between a case's score and the score of a randomly chosen member of the control population (Crawford et al., 2010).

## Results

### Quantitative analyses

The results of the (working) memory subtests displayed in Table 1 show that L obtained scores below the 10th percentile on visuospatial short-term memory and visuospatial working memory. These scores indicate that L has a deficit in visuospatial abilities. The results indicate no deficit in verbal abilities. L obtained average scores on both the verbal working memory and verbal short-term memory subtests. The below average score on digit recall can be explained by the fact that this subtest involved numbers, which is her main weakness. In contrast to L's results, the control group scored within the average range (i.e., 25–75 percentile) on both visuospatial and verbal short-term memory, showing that the memory capacity of the control group is representative for the memory ability of the TD population.

**Table 1:** **Working memory scores of the child with dyscalculia and the control group**.

**Scale**	**Subtest**	**Percentile**
**CHILD WITH DYSCALCULIA (*n* = 1**)		
Verbal short-term memory	Non-word recall	22
	Digit recall	8
Verbal working memory	Listening recall	77
Visuospatial short-term memory	Dot matrix	1
Visuospatial working memory	Odd-one-out	9
**CONTROL GROUP CHILDREN (***n*** = **10**)**		
Verbal short-term memory	Non-word recall	48.1
Visuospatial short-term memory	Dot matrix	66.9

For the control group, the average percentile score per task is displayed.

**Figure 3:**
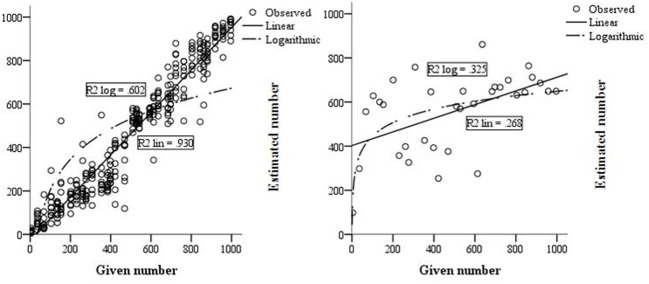
**Linear and logarithmic fit of L (right) and the control group (left) on the 0–1000 number line task**.

The results on the number line tasks displayed in Table 2 show that L's performance on the symbolic and the non-symbolic number line tasks was poor compared to the control group. On all symbolic tasks, her logarithmic and linear *R*^2^-scores explained significantly less variance than in the control group, indicating that L has poor symbolic magnitude representations. Moreover, a comparison of the logarithmic and linear *R*^2^ shows that L's number estimations on the non-symbolic 0–100 and symbolic 0–1000 number line tasks were better described by a logarithmic function than by a linear function, although the proportion of explained variance of L's *R*^2^_log_-score on the 0–1000 number line is still considered very low (Table 2). In comparison, all linear *R*^2^-scores of the control group showed a higher amount of explained variance, indicating good symbolic representations. In addition, the individual fit measures (see appendix) indicate that the number estimations of all children in the control group were better described by a linear function than a logarithmic function on the 0–1000 number line, which is considered age appropriate. As an illustration, the relations between the given numbers and the estimated numbers on the 0–1000 number line task of L and the control group, as represented by a linear and logarithmic regression line, are displayed in Figure 3. The *R*^2^-scores on the symbolic tasks indicate that L's performance deteriorated more than the performance of the control group when magnitudes became larger. Although she already showed lower performance than the control group on the symbolic 0–100 number line, her performance on the 0–1000 number line was even worse, as indicated by a higher mean percent absolute error and lower linear fit.

**Table 2:** **Summary of the results on the non-symbolic and symbolic number line tasks, number of trials, logarithmic and linear fit (*R*^2^), and percent absolute error of the child with dyscalculia and the control group including *p*-values and effect sizes**.

**Number line**	***n***	**Child with dyscalculia (***n*** = **1**)**			**Control group (***n*** = **10**)**			***R*^2^_lin_**		***R*^2^_log_**		**Absolute error**	
		***R*^2^_lin_**	***R*^2^_log_**	**Absolute error (%)**	***R*^2^_lin_**	***R*^2^_log_**	**Absolute error (%)**	***p*-value**	***z*_***cc***_**	***p*-value**	***z*_***cc***_**	***p*-value**	***z*_***cc***_**
**NON-SYMBOLIC**													
0–100	33	0.699	0.712	17.7	0.783	0.695	13.0	.143	−1.19	.454	0.13	.147	1.17
**SYMBOLIC**													
0–100	33	0.849	0.698	10.7	0.955	0.780	4.3	.015	−2.70	.002	−4.10	.000	5.77
0–1000	33	0.268	0.325	20.1	0.930	0.602	5.8	.000	−22.07	.000	13.85	.000	13.75

n, number of trials, excluding two practice trials; R^2^_lin_ and R^2^_log_ represent the linear and logarithmic fit of the estimated numbers; Absolute error, mean percent absolute error. Z_cc_, obvious direct analog of Cohen's d, subscript representing “case-controls.”

A comparison of the mean percent absolute error between L and the control group shows that L's estimations were significantly more distanced from the given number than the control group's estimations (Table 2). All children in the control group showed a lower mean percent absolute error on both symbolic number line tasks than L. Hence, as expected, L's number line estimates were less accurate than the estimates of the TD children.

### Qualitative analyses

#### Processing strategies

For the qualitative analysis of multi-digit number processing, an attempt was made to discern specific fixation patterns from the eye movements on the symbolic 0–100 and 0–1000 tasks and connect these patterns to one of the processing strategies (i.e., holistic, decomposed parallel, and decomposed sequential). Trials were coded as *other* when a fixation pattern was observed but found atypical, as it could not be connected to a specific strategy, and *undefined* when no fixation pattern could be found in the eye movements. With L having severe and specific math problems, it was expected that she would show less strategy use and more atypical or unidentifiable sequences of eye fixations on the number line tasks compared to the control group.

On the symbolic 0–100 number line task, L showed equal fixations at both digits, indicating holistic processing, in 26.7% of the cases (i.e., 64, 53, 24, 49, 91, 43, 41, 99). In 13.3% of the cases (i.e., 96, 89, 87, 34) L fixated first at the tens and then briefly at the units, indicating decomposed parallel processing. Decomposed sequential processing was found in 10% of the cases (i.e., 72, 28, 60), as indicated by fixations at the tens and (almost) not at the units. In addition, also in 10% of the cases, L showed an atypical fixation pattern (i.e., 10, 37, 57) with eye movements starting at the smallest entity and then progressing toward the largest entity. However, in 40% of the cases no fixation pattern could be distinguished (i.e., 78, 18, 27, 83, 46, 14, 61, 74, 66, 32, 19, 80). L fixated at the middle, tens, and units separately in several differing sequences. The percentages for the control group are displayed in Table 3, illustrating the differences in strategy use. L used an atypical strategy about as often as the control group, but her strategy use was significantly more often undefined than the control group average (Table 3). When looking at the type of two-digit numbers, it is not possible to identify a pattern of which numbers are processed in what way, or differences between L and the control children in what strategies are used for the estimation of certain numbers.

**Table 3:** **Overview of the processing strategies used (in percentages) by the child with dyscalculia and the control group (including range of percentages) in the symbolic 0–100 and 0–1000 number line tasks including *p*-values and effect sizes**.

**Strategy**	**Child with dyscalculia**		**Control group^a^**		**Test results**			
	**0–100**	**0–1000**	**0–100**	**0–1000**	**0–100**		**0–1000**	
	**%**	**%**	**%**	**%**	***p*-value**	***z*_***cc***_**	***p*-value**	***z*_*cc*_**
Holistic	26.7	0.0	49.7 (30.0–73.3)^b^	22.0 (0.0–56.7)^b^	.057	−1.84	.144	−1.18
Decomposed parallel	13.3	20.0	21.3 (10.0–46.7)^b^	55.3 (20.0–76.7)^b^	.286	−0.62	.067	−1.72
Decomposed sequential	10.0	0.0	11.0 (6.7–23.3)^b^	1.7 (0.0–10.0)^b^	.461	−0.11	.314	−0.53
Other	10.0	23.3	12.0 (0.0–30.0)^b^	15.0 (3.0–30.0)^b^	.421	−0.22	.184	0.99
Undefined	40.0	56.7	6.0 (0.0–13.3)^b^	6.0 (0.0–26.7)^b^	.000	6.59	.000	5.90

a n = 10, numbers represent the mean percentage of the 10 control children.

b Range of the percentages of the 10 control children, see appendix for individual results of the control children. Z_cc_, obvious direct analog of Cohen's d, subscript representing “case-controls.”

On the symbolic 0–1000 number line task, L showed in 0% of the cases a holistic processing or decomposed sequential processing strategy. L used a decomposed parallel processing strategy in 20% of the cases (i.e., 684, 354, 385, 958, 996, 763) and an atypical strategy in 23.3% of the cases (i.e., 594, 613, 844, 862, 723, 919, 201), with eye fixations starting at the smallest entity and then progressing toward the largest entity. Moreover, in 56.7% of the cases, no specific fixation pattern could be identified (i.e., 422, 261, 528, 542, 398, 510, 804, 277, 469, 230, 104, 153, 697, 135, 880, 308, 636). The eye movements were alternating several times between the middle, hundreds, tens, and units, and while lugging the lever randomly across the number line, L constantly kept checking the separate digits. As a result, it was not possible to identify a (functional) strategy in two thirds of the cases. The percentages for the control group are displayed in Table 3. Again, L's processing of three-digit numbers was significantly more often unidentifiable than in the control group.

#### Estimation strategies

**Figure 4:**
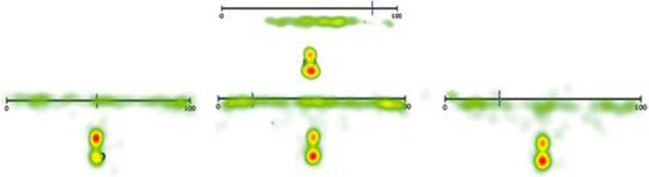
**Heat maps displaying a summary of all eye fixations of L (top) and three randomly chosen control children (bottom) in the 0–100 symbolic number line task**.

Eye movement patterns of the symbolic 0–100 and 0–1000 number line trials were analyzed in a qualitative way to discern information on L's strategy use in estimating numbers on a number line. Eye-tracking videos of the control children were also viewed and coded. Trials were coded as *counting-up* when a child used the beginning of the line as a reference point and counted up from there, and *counting-down* when the end of the line was used as a reference point. Trials in which the child used the midpoint of the line and counted up or down from there were coded as *midpoint*. Finally, trails were coded as *undefined* when no clear estimation strategy could be determined from the eye movement patterns, for example because no reference point was used. If an estimation strategy was evaluated as inadequate, for example when a child used the endpoint strategy for number 18, that trial was double coded as *dysfunctional*. It was expected that L, having severe mathematical problems, would show more dysfunctional and undefined strategies than the control group, as she might make less use of reference points and gaze more at the reference point in the middle of the line (see Van't Noordende and Kolkman, 2013). On the symbolic 0–100 task, L used the beginning of the line as a reference point in 24.2% of the cases (i.e., 10, 3, 43, 9, 72, 32, 28, 5). For the numbers 43, 72, and 32, this strategy was coded as dysfunctional. She used the *midpoint strategy* in 30.3% of the trials (i.e., 53, 46, 14, 24, 74, 66, 87, 41, 57, 60) of which 14, 24, and 87 were estimated with a dysfunctional strategy. The numbers 78, 96, 91, 80, and 99 were all estimated in a functional manner with the *counting-down* strategy. L's eye movement patterns did not reflect an identifiable strategy in 30.3% of the cases (i.e., 18, 27, 64, 83, 49, 61, 89, 37, 34, 19). Instead, her eyes rapidly went back and forth across the number line without seemingly entailing a clear goal. L used a dysfunctional strategy in 26.1% of the identifiable trials. Table 4 illustrates the differences in strategy use between L and the control group: it becomes clear that L's strategy use was significantly more often dysfunctional than the control group average. These results can be summarized in a graphical display, called a heat map. Figure 4 summarizes all eye fixations of L (top) and three randomly chosen control children (bottom) during the symbolic 0–100 task in these heat maps. L's graph perfectly illustrates the randomness of her strategy use, whereas the maps of the control children consistently show the three distinguishable reference points (begin, middle, end).

**Table 4:** **Overview of the estimation strategies used (in percentages) by the child with dyscalculia and the control group (including range of percentages) in the symbolic 0–100 and 0–1000 number line tasks including *p*-values and effect sizes**.

**Strategy**	**Child with dyscalculia**		**Control group^a^**		**Test results**			
	**0–100**	**0–1000**	**0–100**	**0–1000**	**0–100**		**0–1000**	
	**%**	**%**	**%**	**%**	***p*-value**	***z*_*cc*_**	***p*-value**	***z*_*cc*_**
Counting-up	24.2	3.0	22.4 (15.2–33.3)^b^	24.8 (12.1–36.4)^b^	.397	0.28	.016	−2.67
Midpoint	30.3	67.0	36.4 (21.2–42.4)^b^	31.5 (15.2–45.5)^b^	.193	−0.96	.002	3.87
Counting-down	15.2	9.1	25.1 (12.1–33.3)^b^	23.9 (12.1–39.4)^b^	.097	−1.48	.053	−1.88
Undefined	30.3	21.2	18.8 (6.1–42.3)^b^	19.1 (3.0–36.4)^b^	.193	0.59	.421	0.21
Dysfunctional	26.1	42.3	8.8 (0.0–19.4)^b^	11.1 (4.0–21.9)^b^	.010	2.97	.007	3.16

a n = 10, numbers represent the mean percentage of the 10 control children.

b Range of the percentages of the 10 control children, see appendix for individual results of the control children. Z_cc_, obvious direct analog of Cohen's d, subscript representing “case-controls.”

**Figure 5:**
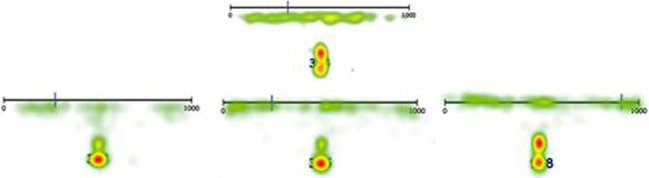
**Heat maps displaying a summary of all eye fixations of L (top) and three randomly chosen control children (bottom) in the 0–1000 symbolic number line task**.

For the symbolic 0–1000 task, L seldom employed the *counting-up* and *counting-down* strategy. However, as was expected, she utilized the middle reference point in two thirds of the trials (i.e., 261, 528, 542, 398, 594, 510, 277, 469, 684, 844, 104, 68, 385, 153, 723, 697, 958, 135, 996, 201, 636, 763), though this appeared to be a dysfunctional strategy in 50% of the cases (e.g., 153, 996). Moreover, 21.2% of the trials were coded as *undefined*, as L made no use of any reference points. In total, she used a dysfunctional strategy in 42.3% of the cases, which was significantly more often than the control group (Table 4). This might illustrate a deficit in adapting strategies to a specific target number, making her number line estimates appear “random.” Again, this seemingly random strategy employment can be illustrated by the heat maps summarizing all eye fixations on the symbolic 0–1000 task (Figure 5).

The results show that different number processing as well as estimation strategies can indeed be discerned using eye-tracking data. Moreover, it is possible to find differences in presence or absence of number processing strategies between children with typically developing numerical skills and children with a number processing deficit based on eye-tracking data. Although this study is exploratory in nature, these results provide a first indication of the possible value of eye-tracking data in combination with number line tasks to discriminate between typical and atypical numerical development in children in future diagnostic procedures.

## Discussion

In this study, the eye movements of a child (L) with DD on a series of number line tasks were compared to those of TD children, in order to determine whether a combination of number line tasks and eye-tracking data can be used to discriminate between TD children and children with a number processing deficit. This case study has provided some important insights in the (non-)numerical processing strategies of children with DD in contrast to TD children. Performance characteristics of L quantitatively and qualitatively differed from the TD children, as there was a high discrepancy in accuracy and strategy use between L and the control group. This evidence suggests that eye-tracking data in combination with number line estimation tasks might be a promising tool in diagnosing dyscalculia in children.

The hypothesis stating that the eye-tracking data of L would indicate that her number representations were logarithmic rather than linear was partly confirmed by the data. In contrast to the 0–100 non-symbolic and 0–1000 symbolic task, her number representations on the 0–100 symbolic line seemed linear rather than logarithmic. The estimations of the control children were better explained by a linear fit on all tasks. L also displayed significantly lower accuracy in estimating numbers than the control group, meaning that her estimations were more distanced from the target number. These results are consistent with her diagnosis dyscalculia. In line with previous research, it might be concluded that the number representations of a child with dyscalculia around age 10 are less precise and therefore similar to younger children (Piazza et al., 2010). However, these results partly contradict findings of Van't Noordende and Kolkman (2013), who investigated number line estimations in children with math disabilities and concluded that number representations of both atypically and a TD children were better represented by a linear fit.

The second hypothesis, stating that L would show more atypical and more unidentifiable fixation patterns than the control group when processing two-digit and three-digit numbers, was largely confirmed. L showed 65% more atypical strategies than the control group on the 0–1000 number line task. In addition, L showed significantly more unidentifiable fixation patterns on the symbolic 0–100 task and nine times more unidentifiable fixation patterns on the 0–1000 task. These results provide important insights in the quality of magnitude representations in children with and without a specific number processing deficit. Eye-tracking data can be a useful tool to visualize the way a child processes multi-digit numbers and therefore creates the opportunity to take a step forward toward a diagnostic process based on cognitive rather than behavioral measures, or at least a combination of both. Because of the exploratory nature of this study, it is not possible to make any statements about which processing strategies indicate typical or atypical development. Future research is needed in order to explore whether TD children, for example, make more use of holistic processing than children with dyscalculia. It can, however, be stated that L made less use of identifiable strategies. Moreover, the relatively high percentages of trials that were coded as “other” in both L and the control group indicate that there might be another functional strategy that can be used to process multi-digit numbers. Future research could shed more light on the functionality and overlap or discrimination between different number processing strategies.

Finally, the third hypothesis stated that L would show more dysfunctional and more undefinable strategies than the control group when estimating numbers on a number line. This was also largely confirmed by the data. L exhibited a dysfunctional strategy significantly more often than the control group and also made less use of reference points in the symbolic 0–100 line (i.e., undefined strategy). Moreover, in the symbolic 0–1000 task, she gazed significantly more often (in 67% of the cases) at the midpoint of the line than the control group. These results are in line with previous research on children with math difficulties (Van't Noordende and Kolkman, 2013). The findings implicate that a child with dyscalculia might have more difficulty with flexibly adapting strategies to the target number than TD children, due to a deficit in the ability to connect a number symbol to a non-symbolic magnitude (i.e., mapping deficit; Dehaene, 2001). The visuospatial memory deficit that was found in L might also partly explain her lower performance on the number line tasks used in this case study (Kolkman et al., under review).

Results of this study have to be interpreted in the light of some methodological caveats. For example, the number line tasks and eye-tracking methodology used in this study have not yet been validated. Hence, it might be that the number sense tasks or the eye-tracking data measure other aspects (i.e., measurement error) in addition to actual competence (Schneider et al., 2008). Moreover, in a small percentage of the total trials (0.5%) calibration errors occurred. This comes to pass when the eye movements are not monitored correctly by the computer and do not appear in the eye-tracking video. Consequently, these trials had to be coded as unidentifiable. Future research focusing on the validity and reliability of these measures (i.e., both eye-tracking methodology and number sense tasks) is essential.

Another caveat relates to the “*n* = 1” nature of this study: only one child with a numerical processing deficit was included. Accordingly, no firm conclusions can be drawn from these data. Future research with a larger empirical sample should investigate the eye fixation patterns in other children with dyscalculia, especially since several subtypes of math disabilities have been described (Wilson and Dehaene, 2007; Rubinsten and Henik, 2009). As a consequence, it might become possible to include eye-tracking measures and number line tasks as an additional tool in the diagnostic process of dyscalculia in the future. This would be of great value, since most current diagnostic instruments are only focused on the behavioral level.

Further theory development on number processing and estimation strategies is needed since a relatively high percentage of the trials in the present study was coded as “other” or “undefined.” The nature of the differences in unidentifiable strategies between L and the control children is not clear. The existence of unidentifiable eye fixation patterns indicates that additional, yet to be defined, strategies might exist. For example, concerning the control group, absence of strategy use but quick estimation might indicate a certain level of automation: a child possesses a precise representation in long term memory of where some of the numbers, e.g., 50, belong on the number line and therefore does not need to employ a specific decoding strategy.

## Conflict of interest statement

The authors declare that the research was conducted in the absence of any commercial or financial relationships that could be construed as a potential conflict of interest.

## Appendix

**Table A1:** **Overview of the individual working memory scores of the child with dyscalculia and the control children**.

	**Dot matrix**			**Non-word recall**		
	**Raw score**	**Standard score**	**Percentile**	**Raw score**	**Standard score**	**Percentile**
L	–	63	1	–	89	22
**CONTROL CHILD**						
1	21	104	58	16	117	85
2	21	94	32	15	106	64
3	28	126	96	12	93	29
4	24	104	55	11	88	19
5	23	103	53	15	106	64
6	30	135	99	13	97	39
7	18	81	11	13	97	39
8	29	130	98	13	97	39
9	32	144	100	15	106	64
10	24	108	67	13	97	39

The standard scores have a mean of 100 and a standard deviation of 15.

**Table A2:** **Overview of the individual logarithmic and linear fit measures of the child with dyscalculia and the control children on the non-symbolic 0–100 and symbolic 0–100 and 0–1000 number line tasks**.

	**NS 0–100**		**S 0–100**		**S 0–1000**	
	**Linear**	**Logarithmic**	**Linear**	**Logarithmic**	**Linear**	**Logarithmic**
L	0.688	0.705	0.847	0.698	0.268	0.325
**CONTROL CHILD**						
1	0.868	0.799	0.848	0.752	0.854	0.600
2	0.748	0.848	0.973	0.796	0.967	0.626
3	0.740	0.634	0.960	0.764	0.947	0.584
4	0.794	0.619	0.975	0.784	0.936	0.592
5	0.696	0.629	0.964	0.773	0.944	0.585
6	0.892	0.816	0.980	0.800	0.934	0.557
7	0.818	0.691	0.975	0.791	0.967	0.629
8	0.783	0.727	0.976	0.787	0.964	0.640
9	0.935	0.776	0.972	0.815	0.887	0.607
10	0.922	0.765	0.963	0.775	0.943	0.620

**Table A3:** **Overview of the individual mean percent absolute errors of the child with dyscalculia and the control children on the non-symbolic 0–100 and symbolic 0–100 and 0–1000 number line tasks**.

**Number line**	**Absolute error (%)**		
	**NS 0–100**	**S 0–100**	**S 0–1000**
L	17.7	10.7	20.1
**CONTROL CHILD**			
1	10.5	7.0	8.0
2	19.3	3.5	4.2
3	16.9	5.0	6.4
4	11.4	3.9	6.1
5	12.0	5.7	6.4
6	13.8	3.6	6.0
7	15.4	4.5	5.8
8	16.0	3.6	4.9
9	6.3	4.0	7.0
10	8.4	4.5	6.1

**Table A4:** **Overview of the individual percentages of the processing strategies in the symbolic 0–100 number line task of the child with dyscalculia and the control children**.

**Strategy**	**Holistic (%)**	**Decomposed parallel (%)**	**Decomposed sequential (%)**	**Other (%)**	**Undefined (%)**
L	26.7	13.3	10.0	10.0	40.0
**CONTROL CHILD**					
1	40.0	20.0	6.7	30.0	3.3
2	43.3	10.0	20.0	13.3	13.3
3	50.0	20.0	23.3	6.7	0.0
4	53.5	23.3	6.7	6.7	10.0
5	46.7	10.0	16.7	13.3	13.3
6	73.3	23.3	0.0	3.3	0.0
7	60.0	10.0	13.3	16.7	0.0
8	60.0	10.0	23.3	0.0	6.7
9	30.0	40.0	0.0	23.3	6.7
10	40.0	46.7	0.0	6.7	6.7

**Table A5:** **Overview of the individual percentages of the processing strategies in the symbolic 0–1000 number line task of the child with dyscalculia and the control children**.

**Strategy**	**Holistic (%)**	**Decomposed parallel (%)**	**Decomposed sequential (%)**	**Other (%)**	**Undefined (%)**
L	0.0	20.0	0.0	23.3	56.7
**CONTROL CHILD**					
1	33.3	56.7	0.0	10.0	0.0
2	36.7	40.0	3.3	16.7	3.3
3	0.0	70.0	0.0	3.3	26.7
4	56.7	20.0	0.0	13.3	10.0
5	6.7	73.3	0.0	6.7	13.3
6	13.3	73.3	0.0	13.3	0.0
7	23.3	66.7	0.0	10.0	0.0
8	36.7	26.7	10.0	23.3	3.3
9	13.3	50.0	3.3	30.0	3.3
10	0.0	76.7	0.0	23.3	0.0

**Table A6:** **Overview of the individual percentages of the estimation strategies in the symbolic 0–100 number line task of the child with dyscalculia and the control children**.

**Strategy**	**Counting-up (%)**	**Midpoint (%)**	**Counting-down (%)**	**Undefined (%)**	**Dysfunctional (%)**
L	24.2	30.3	15.2	30.3	26.1
**CONTROL CHILD**					
1	18.2	30.3	24.2	27.3	8.3
2	30.3	30.3	33.3	6.1	19.4
3	18.2	36.4	33.3	12.1	13.8
4	27.3	27.3	27.3	15.2	0.0
5	15.2	39.4	12.1	33.3	4.5
6	18.2	42.4	21.2	18.2	14.8
7	18.2	21.2	18.2	42.3	10.5
8	27.3	39.4	24.2	9.1	10.0
9	33.3	33.3	27.3	6.1	3.2
10	18.2	33.3	30.3	18.2	7.4

**Table A7:** **Overview of the individual percentages of the estimation strategies in the symbolic 0–1000 number line task of the child with dyscalculia and the control children**.

**Strategy**	**Counting up (%)**	**Midpoint (%)**	**Counting-down (%)**	**Undefined (%)**	**Dysfunctional (%)**
L	3.0	67.0	9.1	21.2	42.3
**CONTROL CHILD**					
1	12.1	30.3	27.3	30.3	13.0
2	30.3	30.3	27.2	12.1	6.9
3	21.1	30.3	12.1	36.4	14.3
4	24.2	30.3	21.2	18.2	4.0
5	33.3	15.2	39.4	12.1	4.8
6	33.3	21.2	24.2	21.2	15.4
7	21.2	39.4	18.2	21.2	11.5
8	21.2	42.4	24.2	12.1	10.3
9	36.4	45.5	15.2	3.0	21.9
10	15.2	30.3	30.3	24.2	14.3
